# Strict adherence to evidence-based protocol in choice of implants and surgical technique leads to fewer hip fracture reoperations

**DOI:** 10.1371/journal.pone.0210239

**Published:** 2019-01-07

**Authors:** Elvira R. Flikweert, Ronald L. Diercks, Gerbrand J. Izaks, Klaus W. Wendt, Martin Stevens, Inge H. F. Reininga

**Affiliations:** 1 Department of Surgery-Traumatology, University of Groningen, University Medical Center Groningen, Groningen, the Netherlands; 2 Department of Orthopedics, University of Groningen, University Medical Center Groningen, Groningen, the Netherlands; 3 University Center for Geriatric Medicine, University of Groningen, University Medical Center Groningen, Groningen, the Netherlands; Public Library of Science, UNITED KINGDOM

## Abstract

**Background and purpose:**

Surgery for hip fractures is frequently followed by complications that hinder the rehabilitation. Only part of the complications are surgery-related, however these, including reoperation may have the highest impact. Operative protocols are designed to treat all patients equally, according to evidence based guidelines. Aim of this study was to investigate the association between strict adherence to an operative protocol and postoperative complications, especially reoperations.

**Materials and methods:**

A retrospective analyses of a prospective cohort. The cohort included all patients aged ≥60 treated for a hip fracture at University Medical Center Groningen between July 2009 and June 2013. The files of the patients were searched for complications, including reoperations. To evaluate adherence to the operative protocol all X-rays were retrospectively reviewed and the fracture type was reclassified. This retrospective fracture classification was compared with the treatment method used. Logistic regression analyses were used to assess whether patients that were not treated strictly according to the operative protocol have higher odds of developing a complication or of undergoing a reoperation.

**Results:**

The study population consisted of 479 patients with a mean age of 78.4 (SD 9.5) years. Reoperation was performed in 11% of the patients during the follow-up period. The operative protocol was not followed strictly in 12% of the patients. When the operative protocol was not followed, the odds of having a reoperation was 2.41 times higher (p = 0.02). The overall complication rate was 75% and did not differ in both groups.

**Conclusion:**

Strict adherence to an evidence-based operative protocol is of major importance toward preventing implant-related problems and reoperations

## Introduction

Hip fractures, including femoral neck fractures and trochanteric proximal femur fractures, are frequently encountered, the typical patient being frail elderly women with multiple comorbidities. A wide variety of treatment options for hip fractures exists; multiple types of materials are used during surgery. Several meta-analyses on the preferred treatment options for each type of fracture have been published [1–3]. However, because of lack of evidence on which surgical method is superior for operating each type of hip fracture, treatment depends upon local preferences [4]. In 2008, a Dutch guideline on the treatment of hip fractures was published [5]. Subsequently, University Medical Center Groningen developed a comprehensive multidisciplinary care pathway for hip fracture patients. This pathway included a protocol guiding the choice of implant and surgical technique, based on this Dutch guideline [6].

Irrespective of treatment method, a significant part of the population treated for a hip fracture will experience a complication in the perioperative period, with complication incidences over 50% reported [7,8]. Medical complications like delirium, pneumonia, heart failure and urine retention are most common [9]. Only some of the complications are related to the surgical procedure, such as wound infections and loss of reduction. These surgery-related complications can however lead to reoperations, and these are adverse effects that have the highest impact on patients, doctors, hospitals and society in terms of costs. These complications caused by the surgical procedure itself might be reduced by strict adherence to an operative protocol. Aim of this study was therefore to investigate the association between strict adherence to the operative protocol and postoperative complications in general, and especially reoperations.

## Materials and methods

### Design

A retrospective analyses of a prospective cohort study was conducted at the departments of Trauma Surgery and Orthopedic Surgery of University Medical Center Groningen (UMCG) in the Netherlands. The procedures employed in this study were approved by the Medical Ethical Committee of University Medical Center Groningen (METc 2011/164). The study was conducted in accordance with the Declaration of Helsinki. Written informed consent was obtained from all patients or their relatives if the patient was not able to give consent.

### Patients

**Fig 1:**
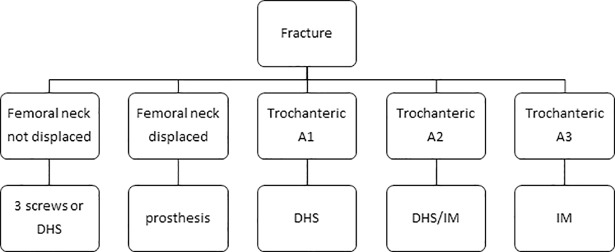
Operative protocol for hip fractures at UMCG. The second row shows the fracture classification; trochanteric fractures are classified according to the AO classification of fractures. Abbreviations: DHS, dynamic hip screw; IM, intramedullary nail.

All patients aged 60 years or older with a hip fracture treated at UMCG between 1 July 2009 and 1 July 2013 were included in this study. Patients were excluded if they had multiple injuries caused by a high-energy trauma. A hip fracture was defined as a femoral neck fracture (dislocated or not dislocated) or a trochanteric fracture (subdivided into type A1, A2 or A3 according to the Arbeitsgemeinschaft für Osteosynthesefragen (AO) Comprehensive Classification). All patients were treated according to the comprehensive multidisciplinary care pathway of UMCG. The operative protocol of this pathway is shown in Fig 1; this protocol defined which implant was indicated for each of the fracture types. In dislocated femoral neck fractures a cemented hemiarthoplasty was placed by a posterolateral incision. In relative young and healthy patients a cemented total hip arthroplasty was placed. All patients got a third generation of cefalosporine for 24 hours as infection prophylactic.

### Data

A case record form was filled in during admittance in the hospital and follow-up in the outpatient clinic of all patients. After the study period the electronic patient files were checked for eventually missed complications occurring between emergency room admittance until the outpatient visit six months after surgery. Data of all departments of the hospital were available. Registered baseline characteristics were demographic information, medical history, fracture classification, trauma mechanism, living situation, and American Society of Anesthesiologists (ASA) classification to get an impression of the extent of co-morbidity [10]. During hospitalization type of implant, waiting time to surgery, operation time, type of anesthesia (general or spinal) and length of hospital stay were recorded from the electronic hospital registration system. Clinical complications and side effects were registered; every unintentionally negative event happening to a patient was registered as a complication. All complications, including reoperation, were registered up to six months postoperatively. The causes of all reoperations were registered.

To evaluate adherence to the operative protocol (Fig 1), all X-rays were retrospectively reviewed and the fracture type was reclassified independently by two researchers. When their conclusions did not match, another trauma surgeon was consulted (KW) to obtain consensus. This retrospective fracture classification was compared with the treatment method used. No strict adherence to the operative protocol was regarded as a risk factor for postoperative complications, especially surgical ones.

### Statistical analysis

All statistical analyses were performed using SPSS Statistics for Windows (Version 22.0, Armonk, NY: IBM Corp.). Descriptive statistics (means, frequencies) were used to describe the characteristics of the study population at baseline and the number and type of complications. To investigate differences in baseline characteristics and complication incidence between patients that were treated strictly according to the operative protocol and those that were not, the Chi-Square test was used for categorical data and the independent sample t-test or, if appropriate, the Mann-Whitney U-test for continuous variables. Additionally, logistic regression analyses were performed to assess whether patients that were not treated strictly according to the operative protocol have higher odds of developing a complication (irrespective of type) or of undergoing a reoperation. All baseline characteristics (such as age, gender, ASA classification, fracture type and prefracture living situation) were assessed for possible confounding. For analysis purposes, age was categorized into age ≤65, 66–75, and >75. ASA classification was also categorized into three categories: ASA 1 and 2, ASA 3, and ASA 4. A variable was considered a significant confounder when the regression coefficient of the variable “protocol adherence” changed more than 10%. A stepwise forward selection method was used. P<0.05 was considered to indicate statistical significance.

## Results

The study cohort consisted of 479 patients who were treated for a hip fracture at UMCG. Another 61 patients admitted for a hip fracture refused to sign the inform consent and were not included. Baseline characteristics are presented in Table 1. Reoperation was performed in 52 patients (11%) during the follow-up period; 20 of these reoperations were performed because of deep infections, 10 because of implant dislocation, nine because the osteosynthesis in patients with a femoral neck fracture had to be replaced by an arthroplasty, and four because of prosthetic dislocation. The total number of complications related directly to the surgical procedure was 63 (13% of the patients). These were wound problems, infection and persistent fluid leakage, (N = 43, 9%) and implant-related problems such as dislocation or breakage (N = 20, 4%). Overall, a total of 359 patients (75%) suffered one or more complications (general medical and surgical) during the six months of follow-up; 210 patients (44%) suffered multiple complications (Table 2). Only 119 patients (25%) did not experience any negative side effect of the treatment.

**Table 1:** Characteristics of the study population (N = 479).

		Total	Protocol adherence	No protocol adherence	P-value
Age (years)^a^		78 (9.5)	79 (9.2)	75 (10.2)	0.01
Gender^b^					0.53
	Male	159 (33)	138 (33)	21 (36)	
	Female	320 (67)	284 (67)	36 (64)	
ASA classification^b^					0.44
	1	28 (6)	24 (6)	4 (7)	
	2	173 (36)	149 (36)	24 (42)	
	3	246 (52)	218 (52)	28 (49)	
	4	28 (6)	27 (7)	1 (2)	
Prefracture living situation^b^					0.34
	Independently	181 (40)	155 (39)	26 (50)	
	Independently, with help of others	129 (29)	114 (29)	15 (29)	
	Assisted living facility	72 (17)	67 (17)	5 (10)	
	Nursing home	68 (15)	62 (16)	6 (12)	
Fracture type^b^					0.16
	A1	55 (12)	47 (11)	8 (4)	
	A2	107 (22)	100 (24)	7 (12)	
	A3	57 (12)	53 (13)	4 (7)	
	Femoral neck, undisplaced	55 (11)	48 (11)	7 (12)	
	Femoral neck, displaced	205 (43)	174 (41)	31 (54)	
Type of anesthesia^b^					0.76
	Spinal anesthesia	168 (35)	149 (35)	19 (33)	
	General anesthesia	310 (65)	272 (65)	38 (67)	
Days to first procedure^b^					0.55
	<1 day	435 (91)	382 (91)	53 (93)	
	≥ 1 day	44 (9)	40 (9)	4 (7)	
Type of implant^b^					<0.001
	Total Hip arthroplasty	31 (7)	28 (7)	3 (5)	
	Hemiarthroplasty	153 (32)	146 (35)	7 (12)	
	Dynamic Hip Screw	134 (28)	106 (25)	28 (49)	
	Intramedullary nail	137 (29)	128 (30)	9 (16)	
	Cannulated screws	24 (5)	14 (3)	10 (18)	
Operation time (minutes)^c^		92 (30–310)	95 (30–298)	84 (40–310)	0.03
Hospital stay (days)^c^		7 (1–120)	7 (1–120)	7 (2–68)	0.05

Data presented as

a mean (SD)

b N (%)

c median (range).

**Table 2:** Results of logistic regression analysis of reoperation.

	Regression coefficient	P-value	Odds ratio (95% CI)
**Protocol adherence**^**a**^	0.88	0.02	2.41 (1.13–5.13)
**Prefracture living situation**^**b**^			
Living independently, with help of others	0.01	0.98	1.01 (0.50–2.01)
Assisted living facility	-1.62	0.03	0.20 (0.05–0.87)
Nursing home	0.01	0.98	1.01 (0.42–2.42)
**Fracture type**^**c**^			
Trochanteric fracture, type A2	-0.11	0.88	0.90 (0.24–3.42)
Trochanteric fracture, type A3	0.40	0.58	1.49 (0.37–6.03)
Femoral neck fracture, undisplaced	0.42	0.54	1.53 (0.40–5.86)
Femoral neck fracture, displaced	0.83	0.14	2.29 (0.75–6.97)

a Reference group: protocol adherence

b Reference category: living independently

c Reference category: trochanteric fracture, type A1

### Adherence to operative protocol

In 422 patients (88%) treatment was done strictly according to the operative protocol, including type of surgery, type of implant and aftercare. In 57 patients (12%) the operative protocol was not followed strictly; these patients had another type of surgery and implant than was indicated by the protocol, the aftercare however was the same as in the protocol group. Significant differences in age, implant, operation time and hospitalization time were found between the two groups (Table 1). Patients that were not treated according to the operative protocol were significantly younger (75 (SD 10) vs 79 (SD 9) years; p = 0.01) and were more frequently treated with osteosynthesis (83 vs 58%; p<0.01)

The incidence of a reoperation was significantly higher in patients whose operative protocol was not strictly followed than in patients who were treated strictly according to the operative protocol (13 patients (23%) vs 39 patients (9%), p = 0.02). Logistic regression analysis showed that when the operative protocol was not followed, the odds of having a reoperation was 2.41 times higher (p = 0.02, Table 2).

Deep surgical infection was the major reason for reoperation (n = 20) There was no statistically significant association between surgical infection and adherence to the surgical protocol. Implant failure was an indication for reoperation in 10 patients: three should have had another implant, had the protocol been adhered to. Biological failure (nonunion or avascular necrosis after osteosynthesis) was the indication for reoperation in nine patients. In seven of these nine patients an arthroplasty should have been performed at the first operation according to the operative protocol.

The total complication rate in both groups was not significantly different (75% vs 75%, p = 0.93, Table 3). Logistic regression analysis showed that, after correction for age, fracture type, prefracture living situation and ASA classification, the odds of having a complication did not differ between the two groups (p = 0.38, Table 4). There was no difference between the two groups in incidence of the most common complications (delirium, heart failure and pneumonia, Table 3). The percentage of surgical complications was slightly higher in the group whose operative protocol was not strictly followed (5% vs 4%), but this was not statistically significant (p = 0.66).

**Table 3:** Incidence of complications during six months of follow-up after surgery for hip fracture.

	Total (N = 479)	Protocol adherence (N = 422)	No protocol adherence (N = 57)
**Number of complications**			
One or more complications	359 (75)	316 (75)	43 (75)
None	120 (25)	106 (21)	14 (12)
Delirium	91 (19)	80 (19)	11 (19)
Pneumonia	47 (10)	41 (10)	6 (11)
Heart failure	25 (5)	21 (5)	4 (7)
Wound problems	43 (9)	35 (8)	8 (14)
Implant-related complication	20 (4)	17 (4)	3 (5)
Reoperation	52 (11)	39 (9)	13 (23)

Data are given as number of complications (N (%)).A patient can have multiple complications.

**Table 4:** Results of logistic regression analysis of developing a complication.

	Regression coefficient	P-value	Odds ratio (95% CI)
**Protocol adherence**^**a**^	0.33	0.37	1.39 (0.68–2.84)
**Prefracture living situation**^**b**^			
Living independently, with help of others	0.001	0.99	1.00 (0.59–1.69)
Assisted living facility	0.50	0.20	1.65 (0.78–3.50)
Nursing home	0.09	0.82	1.09 (0.51–2.33)
**Fracture type**^**c**^			
Trochanteric fracture, type A2	0.63	0.12	1.88 (0.86–4.14)
Trochanteric fracture, type A3	0.87	0.07	2.39 (0.95–6.03)
Femoral neck fracture, undisplaced	0.12	0.79	1.13 (0.48–2.61)
Femoral neck fracture, displaced	0.53	0.13	1.70 (0.85–3.41)
**Age (categorical, in years)**^**d**^			
66–75	-0.29	0.47	0.75 (0.34–1.65)
76–85	0.23	0.58	1.25 (0.56–2.81)
>85	0.30	0.49	1.34 (0.58–3.12)
**ASA classification**^**e**^			
ASA 3	0.36	0.14	1.44 (0.89–2.32)
ASA 4	0.65	0.28	1.92 (0.60–6.17)

a Reference group: protocol adherence

b Reference category: living independently

c Reference category: trochanteric fracture, type A1

d Reference category: up to 65 years

e Reference category: ASA 1–2

## Discussion

Aim of this study was to investigate the importance of adherence to an evidence-based surgical protocol. The results of this study show that strict adherence to an operative protocol for elderly patients with a proximal femur fracture is associated with a reduced number of reoperations. This is consistent with another study that showed a reduction in reoperation rate after hip fracture surgery in a teaching hospital when an algorithm for surgical treatment was strictly followed [11]. It is plausible to assume that this led to better outcomes and lower overall medical costs.

In the comprehensive care pathway at UMCG a strict operative protocol based on scientific evidence was used [5]. The quality of care at this point was acceptable, because 89% of the patients were treated according to the operative protocol. However, the fact that reoperation rates were higher in patients whose operative procedure was not performed according the operative protocol (23% vs 9%) stresses the importance of classifying fractures on the preoperative X-rays properly and adhering to the established operative protocol.

Nonadherence to the operative protocol happened more often with younger patients than older patients. The percentage of patients not treated according to the protocol was higher in patients with a DHS or cannulated screws than in the arthroplasty group. In nine patients osteosynthesis material had to be replaced because of nonunion or avascular necrosis, which was followed by an arthroplasty. In seven of these nine patients an arthroplasty should have been performed at the first operation according to the operative protocol. This suggests that it is the surgeon’s decision to try to save a young patient’s hip. Performing osteosynthesis instead of an arthroplasty in displaced femoral neck fractures in active patients just over age 60 can be a well-advised decision, but the serious risk of reoperation should certainly be discussed with the patient.

Operation time and hospitalization time were shorter in the group that did not adhere strictly to the protocol; this can largely be explained by the fact that performing an osteosynthesis takes less time than an arthroplasty and younger patients can be mobilized quicker.

There was no significant difference between the groups in overall complication rates. This is probably because all patients adhered to the same multidisciplinary pathway, which included consultation with the geriatrician as well as prophylactic antithrombotic and antibiotic measures.

The main limitation of this study is the small sample size of the ‘non-adherence to protocol group’. However this also shows that the protocol is feasible. For the extracapsular fractures the group that was not treated according to the protocol was too small to draw any conclusions. This might be because the protocol for extracapsular fractures is very solid: most surgeons will treat an A1 fracture with a DHS implant and an A3 fracture with intramedullary fixation; in A2 fractures the surgeon can choose either of them.

The reoperation rate might be underestimated since patients may have sought medical care in another hospital. The risk for this was probably small, because it is common in our region to refer patients back to the original treating hospital in case of problems.

Our study supports the hypothesis that strict adherence to an evidence-based operative protocol is of major importance toward preventing implant-related problems and reoperations.

## Supporting information

S1 File Anonymized study data. (SAV)
